# Cognitive-behavioral rehabilitation vs. treatment as usual for bipolar patients: study protocol for a randomized controlled trial

**DOI:** 10.1186/s13063-017-1896-5

**Published:** 2017-03-28

**Authors:** Bernardo Carramão Gomes, Cristiana Castanho Rocca, Gabriel Okawa Belizario, Beny Lafer

**Affiliations:** 1 Department of Psychiatry, University of São Paulo School of Medicine, Rua Dr. Ovídio Pires de Campos, 785, São Paulo, 05403-010 Brazil; 2 Department and Institute of Psychiatry, Bipolar Research Program, University of São Paulo Medical School, Sao Paulo, Brazil; 3 Department and Institute of Psychiatry, Faculdade de Medicina da Universidade de São Paulo (USP), São Paulo/SP, Brazil

**Keywords:** Bipolar disorder, Psychotherapy, Treatment as usual, TAU, Cognitive rehabilitation, Functional impairment, Cognitive functioning, Cognitive-Behavioral Rehabilitation

## Abstract

**Background:**

Bipolar disorder (BD) is commonly associated with cognitive and functional impairments even during remission periods, and although a growing number of studies have demonstrated the benefits of psychotherapy as an add-on to pharmacological treatment, its effectiveness appears to be less compelling in severe presentations of the disorder. New interventions have attempted to improve cognitive functioning in BD patients, but results have been mixed.

**Methods:**

The study consists of a clinical trial comparing a new structured group intervention, called “Cognitive-Behavioral Rehabilitation,” with treatment as usual (TAU) for bipolar patients. The new approach is a combination of cognitive behavioral strategies and cognitive remediation exercises, consisting of 12 weekly group sessions of 90 min each. To be included in the study, patients must be diagnosed with BD type I or II, aged 18–55 years, in full or partial remission, and have an IQ of at least 80. A comprehensive neuropsychological battery, followed by mood, social functioning, and quality of life assessments will occur in three moments: pre and post intervention and 12 months later. The primary outcome of the study is to compare the time, in weeks, that the first full mood episode appears in patients who participated in either group of the study. Secondary outcome will include improvement in cognitive functions.

**Discussion:**

This is the first controlled trial assessing the validity and effectiveness of the new “Cognitive-Behavioral Rehabilitation” intervention in preventing new mood episodes and improving cognitive and functional impairments.

**Trial registration:**

Clinicaltrial.gov, NCT02766361. Registered on 2 May 2016.

**Electronic supplementary material:**

The online version of this article (doi:10.1186/s13063-017-1896-5) contains supplementary material, which is available to authorized users.

## Background

Bipolar disorder (BD) is a severe medical condition often associated with functional impairments even when affected individuals are euthymic [1]. Many studies report cognitive deficits in this population, most often in executive functions [2], attention [3], and verbal memory [4], which is coherent with recent meta-analyses on this topic [5, 6]. Recent studies have also established an association between cognitive deficits and functional impairments [7]. This evidence leads some authors to suggest that bipolar patients should benefit from cognitive rehabilitation strategies similar to those conducted in patients with schizophrenia [8].

Structured psychotherapies for BD associated with standard pharmacotherapy are associated with longer periods of remission, reduction in manic and depressive symptomatology, and faster recovery from episodes when compared to pharmacological treatment alone [9]. The majority of current studies suggest psychotherapy as an add-on to pharmacological treatment, even during euthymic periods [10].

Some studies have reported negative results regarding the effects of psychotherapies in preventing new mood episodes in BD [11]. A multicenter study including 253 patients with BD, in a heterogeneous sample with patients in diverse mood states, identified that Cognitive Behavior Therapy (CBT) prevented new mood episodes only in patients with less than 12 previous episodes [12]. Similar negative results in recovering and preventing mood episodes were found in group psychotherapy settings [13, 14]. The effectiveness of structured approaches most likely depends on the number of previous mood episodes [15]. Most negative studies included more severe presentations of BD, often including patients with more than two co-morbidities or many previous mood episodes; these observations have guided some researchers to delineate the importance of a staging model in BD [16].

Due to the disabling cognitive impairments in BD, new interventions were developed, namely cognitive rehabilitation and functional remediation. The first term describes a cognitive-oriented approach, developed to enhance specific cognitive domains such as attention and executive functioning [17]. The second one claims to enhance these same domains using an ecologic method in day-by-day tasks [18]. Despite such differences, we have chosen to use the term “cognitive remediation” to describe both approaches after considering the lack of evidence in this field. Deckersbach et al. [17] ran an open trial with 18 bipolar patients presenting depressive symptoms. After 14 individual sessions of cognitive remediation, patients demonstrated lower residual depressive symptoms and increased occupational and psychosocial functioning; the results persisted after three months. Another trial, conducted by Torrent et al. [19], evaluated cognitive remediation, delivering 21 weekly sessions in a group format. It consisted of three arms: (1) functional remediation; (2) psychoeducation; and (3) standard pharmacological treatment (TAU). The study included 239 euthymic, type I and type II bipolar patients, employing the Functioning Assessment Short Test (FAST) as the main outcome. Results suggest that psychoeducation and group cognitive remediation were better than TAU in improving functioning. Theory of mind [20] guided a new intervention designed to improve social cognition in BD [21]. The study included 37 patients randomly assigned to 18 group sessions of Social Cognition and Interaction Training (SCIT) or TAU alone. The SCIT group revealed an improvement in emotional perception and a decrease in depressive symptomatology. Lastly, Demant et al. [22] developed a 12-week group intervention of cognitive remediation. Their first trial included 46 BD patients, partially or fully remitted, randomly assigned to either cognitive remediation or standard treatment. The 26-week follow-up revealed no statistical differences in executive function, verbal memory, sustained attention, and psychosocial behavior, despite participants in the cognitive remediation group reporting a significant improvement in verbal fluency and quality of life [23].

These innovative trials encouraged the emerging field of cognitive remediation in BD. Cognitive remediation seems to be a feasible and partially efficacious method for treating residual depressive symptoms and improving functional recovery. Paradoxically, it is unclear whether such interventions are capable of promoting cognitive recovery in BD patients, the core reason for its creation.

Traditionally, cognitive remediation methodologies employ task-focused approaches. However, when testing its efficacy, restrictive settings are often utilized [24], which brings criticism for creating low ecological validity (i.e. the individual shows improvement in a specific task, but does not transfer it into their daily lives); consequently, new approaches are being proposed rather focusing on daily tasks [18].

Current psychological interventions for BD focus on the reduction of mood symptomatology and prevention of new bipolar episodes [25] and although these interventions may secondarily improve cognitive impairments, new psychological approaches enabling BD patients to deal with future episodes and addressing cognitive deficits are desirable.

This study aims to evaluate the effectiveness of a new intervention that combines cognitive rehabilitation and CBT strategies. Cognitive Behavioral Rehabilitation (CBR) was designed in an attempt to create a new intervention that could not only prevent new mood episodes (main outcome) but also improve memory, attention, executive functioning (secondary outcomes), and enhance quality of life (tertiary outcome).

### Hypotheses

The study hypothesizes that CBR, compared with TAU, will:

1. Expand the period of time until the first new episode—our primary outcome measure;
2. Improve attention, mental flexibility, working memory, and emotional recognition—our secondary outcome.

In an exploratory analysis, we will also assess whether CBR:

1. Enhances functional, social skills, and quality of life scores;
2. Increases sleep quality and knowledge about the disorder; and
3. Reduces impulsivity.

## Methods/Design

**Fig. 1:**
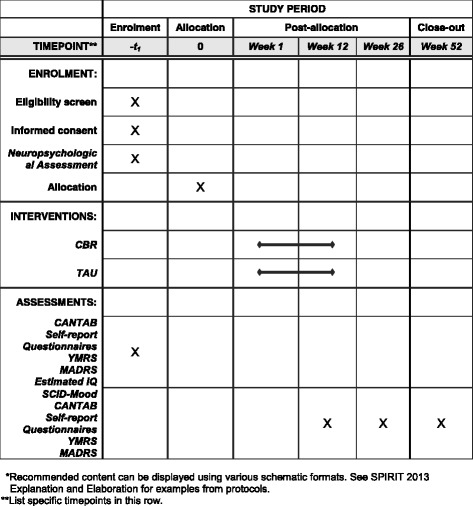
Schedule of enrolment, interventions, and assessments of study

The study compares CBR with TAU, the latter being the commonly offered pharmacological treatment to bipolar patients. The psychological intervention will consist of 12 weekly group sessions, lasting 90 min each, and including eight to ten individuals. Participants will be randomly assigned to one of the two arms and followed for 12 months thereafter (Fig. 1). During the entire study, all patients will be medicated accordingly to their clinical needs and all changes in medication will be recorded, following the Necessary Clinical Adjustment (NCA) instrument. The NCA records medication adjustments implemented to reduce symptoms, improve response and functioning, or handle unbearable side effects [26].

### Participants

In order to be included, patients must be aged 18–55 years, literate, present an IQ score higher than 80, have been diagnosed with bipolar I or II based on Structured Interview for DMS IV (SCID) [27], and be in full or partial remission according to the DSM IV [28]. Exclusion criteria are: presence of substance or alcohol abuse in the last six months; current suicide risk; organic mental disorders; or scores higher than 12 in the Montgomery-Åsberg Depression Rating Scale (MADRS) or the Young Mania Rating Scale (YMRS) at the beginning of the intervention [28].

Recruiting will take place at an outpatient service provided by the Bipolar Disorder Research Program (PROMAN) at the University of São Paulo Medical School, Brazil. Patients will receive invitations individually and sign an informed consent. This research was approved by an internal ethical committee and registered in ClinicalTrials.gov: NCT02766361.

### Procedure and outcomes

Once included, patients will complete the Portuguese versions of the following self-report questionnaires:

- Abbreviated instrument of quality of life (WHOQOL-bref) [29]: evaluating quality of life scored in four domains: physical, psychological social, and environmental
- Barratt Impulsiveness Scale 11 (BIS 11) [30]: assessing the personality/behavioral construct of impulsiveness. It includes 30 items describing common impulsive or non-impulsive behaviors and preferences
- Social Skills Inventory (IHS) [31]: self-report instrument assessing social skills. It consists of 38 items, each describing a situation of interpersonal relationship and a demand for ability to react to that situation
- Biological Rhythms Interview of Assessment in Neuropsychiatry (BRIAN) [32]: consisting of 21 items divided into five main areas related to circadian rhythm disturbances in psychiatric patients, including sleep, activities, social rhythms, eating patterns, and predominant rhythm
- Functioning Assessment Short Test (FAST) [33]: a 24-item interview assessing functional impairments in BD, and including autonomy, occupational functioning, cognitive, financial issues, interpersonal relationships, and leisure time
- Knowledge about Affective Disorders Interview (KADI): brief questionnaire evaluating the patient’s knowledge about prognostic, medication, etiology, symptoms, and diagnosis of BD [34]

A brief neuropsychological battery will be conducted, which includes two subtests of matrix reasoning and vocabulary from the Wechsler Abbreviated Scale of Intelligence (WASI) [35] and following subtests from the Cambridge Neuropsychological Test Automated Battery (CANTAB): Motor Screening Task (MOT); Rapid Visual Information Processing (RVP); Reaction Time (RTI); Spatial Span (SSP); Spatial Working Memory (SWM); One Touch Stockings of Cambridge (OTS); Pattern Recognition Memory (PRM); Delayed Matching to Sample (DMS); Attention Switching Task (AST); and Emotion Recognition Task (ERT).

Participants will also complete the initial assessment and mood module of the Structured Clinical Interview for DSM-IV (SCID-IV) [36] at week 12 after inclusion into the study and 12 months thereafter.

## Interventions

### Treatment as usual (TAU)

The control group from this study will receive standard outpatient treatment offered in our clinic, which involves psychopharmacological mood stabilization and regular contacts with mental health nurses. The type and dosage of pharmacological treatment will follow the physician decision, respecting individual demands. All pharmacological treatment will be monitored and recorded in accordance to the Litmus study [26].

### Cognitive Behavioral Rehabilitation (CBR)

We developed a 12-session intervention combining previous experience in cognitive behavior therapy for bipolar patients [14] with several elements of cognitive remediation. The first step was to identify behaviors that have an important role in patients’ autonomy, followed by determining which cognitive domains are involved. The core objective was to promote the generalization of the learnt behaviors in the daily routine. Described below is the arrangement of each session, divided into three major modules.

The first module comprises four sessions attempting to improve attention and memory, considering the necessity to retain the information discussed throughout the sessions. There are two target behaviors involved: adherence to pharmacological treatment and mood monitoring. The cognitive remediation exercises seek to enhance verbal and visual memories, while secondarily enhancing attention with the paper material included in the manual. In the first session, group members and psychotherapists introduce themselves, followed by a discussion regarding the manual, individual’s expectations, and the importance of attendance. The second session explores the concept of attention and its importance as a door to further cognitive functions; the group also learns exercises aimed at training attention and memory. The third session focuses on medication adherence and its relation to attention. The core of the third session is the organization of the patient’s environment, which is frequently chaotic; a discussion about cues is encouraged at the end of the session. The fourth session starts by introducing mood graphics to patients and the importance of the early identifying of mood episodes. At the end of the first module, patients are encouraged to cook as a method of reinforcing what they have learned while enhancing their autonomy.

The second module targets social cognition and communication. The fifth session familiarizes the patients with the concept of automatic thoughts [37] and a guide to identify its presence. Cognitive distortions are discussed along with examples provided by the participants’ own experiences. The sixth session begins returning to the initial theme by habituating patients to the automatic thought record [38]; patients are stimulated to restructure their own thoughts during experiences identified in previous sessions. Mental flexibility and empathy are introduced and discussed. The seventh session acquaints patients with assertive communication and emotion recognition by teaching role-playing exercises and the importance of positive assertiveness. The eighth session follows the same agenda as the seventh.

The last module of CBR aims at problem-solving strategies and relapse prevention. The ninth session begins with the identification of personal problems, mainly by distinguishing it from preoccupations; the topic is important because patients often incorporate their problems to expectations and desires, generating an urge to abandon them. The session ends by emphasizing the importance of mental flexibility in generating as many responses as possible to each identified problem. In the tenth session, patients learn solving-problem techniques in a systematic setting. The 11th session is devoted to reviewing information and clarifying possible doubts from the patients; patients are also encouraged to debate the importance of regular routines and regular sleep, which can be adjusted using sleep hygiene techniques. A progressive muscle relaxation ends the session. Finally, the last session’s target is to avoid future mood relapses, by returning to the personal goals defined in the first session and prompting patients to develop a prevention plan. The acronym H.U.M.O.R. resumes the core points of the post-intervention maintenance program: (1) Habituate to a regular routine; (2) Use what you have learnt; (3) Monitor your mood; (4) Observe arising problems and deal effectively with it; and (5) Respond to automatic thoughts. All patients in the CBR group will also receive TAU.

## Statistical analysis

### Sample size

The sample size calculation was based on the proportion of patients that remain episode-free after 12 months following a group intervention. Previous studies utilizing TAU exhibited a decrease of bipolar relapses in 30% of patients after a one-year follow-up [39]. The present study anticipates a 55% success rate in prevention of mood relapses, during the same period, in patients assigned to the CBR. Thus, considering an 80% power to obtain a 5% significance, an estimated sample of 28 individuals per group, 56 in total, should be sufficient to achieve significant results. A previous study conducted by the same research team [14] measured a drop-out rate of 10% in a one-year follow-up. For this latter reason, the study will consist of 60 participants.

### Baseline and follow-up data

In order to measure the effectiveness of the intervention, the study will employ the following statistical tests: (1) Chi-squared and Mann–Whitney to test homogeneity between the groups at the beginning of the interventions; (2) Student’s t-test or Mann–Whitney to investigate the effects of such interventions, pre and post treatment, depending on the distribution of the data; (3) an analysis of variance, with and without adjustment for mood symptoms scores, IQ and BD duration, for comparison between groups; and (4) the Kaplan–Meyer survival method with log rank test for statistical analysis, to investigate the survival data between groups, which measures in weeks, the time to the first episode as an event. To be considered as an intervention completer, patients assigned to CBR should attend at least eight sessions (66.7%). Intent to treat analyses will be conducted in order to include all available data and missing data will be handled accordingly to the SPIRIT checklist recommendations (Additional file 1).

## Discussion

The trial compares a new psychological intervention, developed to treat cognitive dysfunction in BD patients, with TAU, in an attempt to investigate its effects in cognitive functioning and its ability to prevent new mood episodes. The objective of this study is to understand the connection between these outcomes and the successful prophylactic treatment of BD. Previous studies with more complex presentations of BD failed to prevent further episodes [13] or to improve cognitive functioning in BD patients [23]. Our hypothesis is that the combination of cognitive behavior therapy and cognitive rehabilitation should provide relevant and lasting improvements, and consequently allow for a better generalization of the improvements to daily routines.

## Limitations

In comparison with previous studies [19], the current study utilizes a shorter intervention, which may be insufficient to effectively deal with many aspects of the disorder. Further experiments, using a larger samples and multicenter settings are necessary if this new intervention proves to be beneficial in our original single site proof-of-concept study.

## Perspectives

We hope that this trial will contribute to a better understanding of the clinical responses for the two different treatment approaches being used in this study; the results may have important clinical implications in the management of BD patients. Previous negative findings may be due to an absence of cognitive rehabilitation strategies in traditional protocols, a limitation we try to overcome in this study by focusing in daily activities, and expecting these behaviors to be more easily translated into clinical improvements and extended periods of remission.

## Trial status

The study is underway and currently includes 40 patients, of which 29 have completed the initial evaluations and 11 are currently in the follow-up period following participation in either CBR meetings or TAU.

## Additional file

Additional file 1: We have included a copy of the SPIRIT checklist. (DOC 119 kb)

## Abbreviations

BD: Bipolar disorder
CBR: Cognitive Behavioral Rehabilitation
CBT: Cognitive Behavioral Therapy
PROMAN: Bipolar Disorder Program
